# Statement of Retraction: Eleutheroside E alleviates cerebral ischemia-reperfusion injury in a 5-hydroxytryptamine receptor 2C (Htr2c)-dependent manner in rats

**DOI:** 10.1080/21655979.2026.2730737

**Published:** 2026-10-01

We, the Editors and Publisher of the journal *Bioengineered*, have retracted the following article from publication.

Liu, Z., Gao, W., & Xu, Y. (2022). Eleutheroside E alleviates cerebral ischemia-reperfusion injury in a 5-hydroxytryptamine receptor 2C (Htr2c)-dependent manner in rats. *Bioengineered*, 13(5), 11718–11731.https://doi.org/10.1080/21655979.2022.2071009

Since publication, significant concerns have been raised by a third party about the integrity of the data and reported results in the article. Specifically, concerns were raised about alleged image duplication with similar images from unrelated articles:

- Figure 1a Control, I/R and I/R+EE panels appear to be duplicated with similar images in Figure 4A Sham, I/R and DZX+I/R panels, respectively, from Lei et al, 2018 (https://doi.org/10.3892/mmr.2018.8925).

- Figure 1c Control and I/R panels appear to be partially duplicated with similar images in Figure 4B Sham x40 and DZX+5-HD+I/R x40 panels, respectively, from Lei et al, 2018 (https://doi.org/10.3892/mmr.2018.8925).

- Figure 2d Caspase 3, Caspase 6 and GAPDH bands appear to be duplicated with similar images in Figure 2b p-S536-p65, p-κBα and κBα bands, respectively, from Wei et al, 2020 (https://doi.org/10.1186/s13020-020-0295-0).

- Figure 5d I/R+EE+siRNA-Htr2c-3 panel appears to be partially duplicated with a similar image in Figure 1d IL-1ß+100 panel from Wei et al, 2020 (https://doi.org/10.1186/s13020-020-0295-0).

- Figure 6b Caspase 6 I/R+EE, I/R+EE+siRNA-NC and I/R+EE+siRNA-Htr2c-3 band appears to be horizontally flipped and duplicated with a similar image in Figure 2d Aggrecan band from Wei et al, 2020 (https://doi.org/10.1186/s13020-020-0295-0).

- Figure 6b Caspase 7 Control, I/R and I/R+EE band appears to be duplicated with a similar image in Figure 2d Coll X band from Wei et al, 2020 (https://doi.org/10.1186/s13020-020-0295-0).

When approached for an explanation, the authors did not respond to our queries and so these serious concerns remain unaddressed.

As verifying the validity of published work is core to the integrity of the scholarly record, we are therefore retracting the article. The authors have been informed of this decision.

We have been informed in our decision-making by our policy on publishing ethics and integrity and COPE guidelines. 

The retracted article will remain online to maintain the scholarly record and will be digitally watermarked on each page as ‘Retracted’. 

